# Malignant Phyllodes Tumor and Acute Megakaryoblastic Leukemia Sharing a Common Clonal Origin

**DOI:** 10.1155/2013/934781

**Published:** 2013-12-17

**Authors:** Yngvar Fløisand, Klaus Beiske, Geir Erland Tjønnfjord, Dag Heldal, Bodil Bjerkehagen, Mona-Elisabeth Revheim, Sverre Heim, Øyvind Sverre Bruland, Kirsten Sundby Hall, Anne Tierens, Jan Delabie

**Affiliations:** ^1^Department of Hematology, University of Oslo, P.O. Box 4950, Nydalen, 0424 Oslo, Norway; ^2^Department of Pathology, University of Oslo, P.O. Box 4950, Nydalen, 0424 Oslo, Norway; ^3^Institute of Clinical Medicine, University of Oslo, P.O. Box 4950, Nydalen, 0424 Oslo, Norway; ^4^Department of Nuclear Medicine, Oslo University Hospital, University of Oslo, P.O. Box 4950, Nydalen, 0424 Oslo, Norway; ^5^Section for Cancer Cytogenetics, Institute for Medical Informatics, University of Oslo, P.O. Box 4950, Nydalen, 0424 Oslo, Norway; ^6^Department of Oncology, University of Oslo, P.O. Box 4950, Nydalen, 0424 Oslo, Norway; ^7^Department of Pathology, Oslo University Hospital, Radiumhospitalet, P.O. Box 4950, Nydalen, 0424 Oslo, Norway

## Abstract

There is a well-known association in male patients between mediastinal germ cell tumors (GCT) and hematologic malignancies, with a propensity towards acute megakaryoblastic leukemia. These rare malignancies have been shown to share a common clonal origin, often deduced from the finding of isochromosome 12p, i(12p), in cells from both the solid tumor and the leukemia, and thus are now known to represent different manifestations of the same clonal process. We treated a young female patient with a malignant phyllodes tumor followed by an acute megakaryoblastic leukemia and found several of the same marker chromosomes by karyotype analysis of cells from both the tumor and the leukemia implying a common clonal origin of the two. To the best of our knowledge, this has not been demonstrated in phyllodes tumors before, but indicates that the same type of leukemization may occur of this tumor as has been described in mediastinal GCT.

Acute megakaryoblastic leukemia (AML M7) is rare, accounting for <5% of all cases of AML [1]. It may occur in specific clinical settings, such as Down's syndrome or in association with mediastinal germ cell tumors (GCT) [2–5]. Nichols et al. and DeMent et al. first reported the association between GCT and hematologic malignancies. Moreover, cytogenetic findings indicated a common clonal origin, and the disease was characterized by an aggressive clinical course [6, 7]. All reported patients were males and had mediastinal localization of the GCT. Here we report a unique case of malignant phyllodes tumor, a rare tumor representing 0.3% to 1% of breast fibroepithelial neoplasia [8, 9], followed by clonally related acute AML M7.

A 20-year-old patient presented with a lump in her left breast. An excision biopsy revealed a malignant phyllodes tumor (Figures 1(a) and 1(b)). The epithelial component was benign, whereas the mesenchymal component was malignant with 19 mitoses per 10 high power fields and focal necrosis. Actin and desmin were expressed, but not MyoD1. Cytogenetic analysis showed a complex karyotype with numerous chromosomal aberrations (Table 1). Treatment consisted of radical excision of the tumor followed by adjuvant chemotherapy with six courses of doxorubicin (60 mg/m²) and ifosfamide (6 g/m²). Nine months after diagnosis, thrombocytopenia was diagnosed. A CT scan revealed osteolytic lesions in the manubrium sterni and lesions in the liver. The patient rapidly developed pancytopenia and rising levels of lactate dehydrogenase and transaminases. Metastatic disease from the malignant phyllodes tumor was presumed. However, flow cytometry and biopsy of the bone marrow biopsy revealed AML M7 (Figures 1(c)–1(f)). The leukemic cells were CD61+, FVIII+, and CD41+ and also expressed CD34 and CD56. There was no expression of CD99, desmin, or cytokeratin. Cytogenetic analysis of leukemic cells revealed several of the same complex karyotypic changes previously detected in the phyllodes tumor, including rearrangements of chromosomes X, 3, 13, 16, and 19 as well as several marker chromosomes. The patient received induction chemotherapy consisting of amsacrine, etoposide, and cytarabine. Fifteen days after the start of chemotherapy, a bone marrow biopsy showed bone marrow necrosis with no evidence of viable tumor. Due to fever she was readmitted and abdominal pain, pancytopenia, elevated LDH, and elevated transaminases subsequently developed. PET/CT showed a diffusely enlarged liver with intense FDG uptake, high uptake in mediastinal lymph nodes, and irregular uptake in the skeleton. A liver biopsy revealed massive infiltration of AML M7. Human chorionic gonadotropin beta was elevated at 187 IU/L. Fulminant liver failure and multiorgan failure rapidly followed to which the patient succumbed. An autopsy was declined.

To further test to what extent the patient's malignant diseases were related, the phyllodes tumor was retrospectively screened with antibodies against megakaryocytic markers. Surprisingly, a small focal area displaying distinct and strong positivity for CD61, FVIII, CD34, and CD31 was identified within the stromal part of the tumor. This part of the tumor showed a cohesive growth pattern with a clear cell appearance that was different from the spindle-shaped cell proliferation seen in other parts of the tumor (Figures 2(a) and 2(b)). Fluorescence (FISH) and chromogenic (CISH) in situ hybridization revealed identical genetic changes in both tumor components: one copy of chromosome 8, 13q14, and 17, but two copies of 14q32 and 16p11 (Figures 2(c)–2(f)). In conclusion, the latter findings, as well as the cytogenetic findings of breast tumor and bone marrow, convincingly indicated that AML M7 originated from the phyllodes tumor and shared its clonal origin.

To the best of our knowledge, this is the first report of AML M7 arising in a malignant phyllodes tumor and showing the same clonal origin. This case shows a striking parallelism with AML in male patients arising in composite mediastinal GCT [6, 7, 10]. In the majority of these patients, leukemia developed less than two years after initial treatment for GCT and in more than seventy percent even within 12 months [4].

Phyllodes tumors are, like GCT, multilineage tumors and heterologous differentiation like osteosarcoma, chondrosarcoma, and liposarcoma known to occur within the stromal area. However, no hematological neoplasia has hitherto been described in phyllodes tumors as opposed to mediastinal GCT and a systematic study of malignant phyllodes tumors might reveal whether hematopoietic differentiation is more common than assumed.

## Figures and Tables

**Figure 1:**
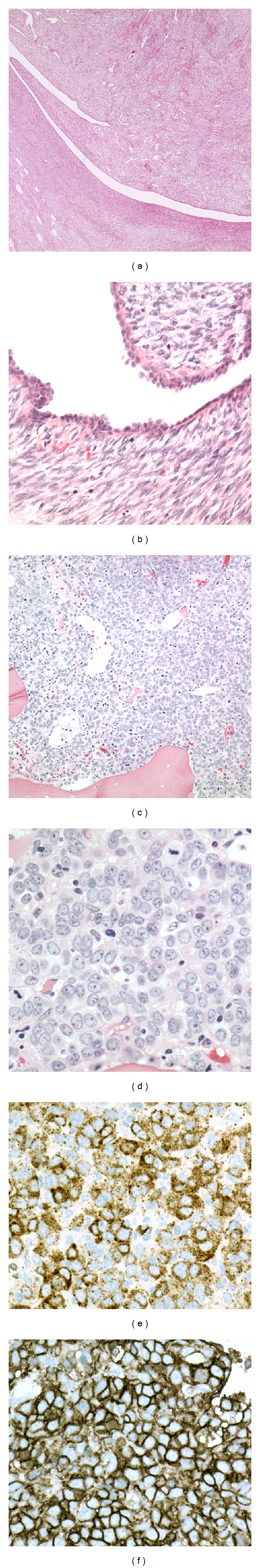
Histology of the malignant phyllodes tumor (panels (a) and (b)) and acute megakaryocytic leukemia (panels (c)–(f)). Hematoxylin and eosin staining of the phyllodes tumor showed a benign epithelial component and a malignant stromal component (panel (a) 20x and panel (b) 200x enlarged). The bone marrow was diffusely infiltrated by large blasts (panels (c) and (d), H&E-stained sections, 100x and 400x enlarged, resp.). The blasts expressed factor VIII and CD31 (panels (e) and (f), resp., immunoperoxidase-stained sections, 400x enlarged).

**Figure 2:**
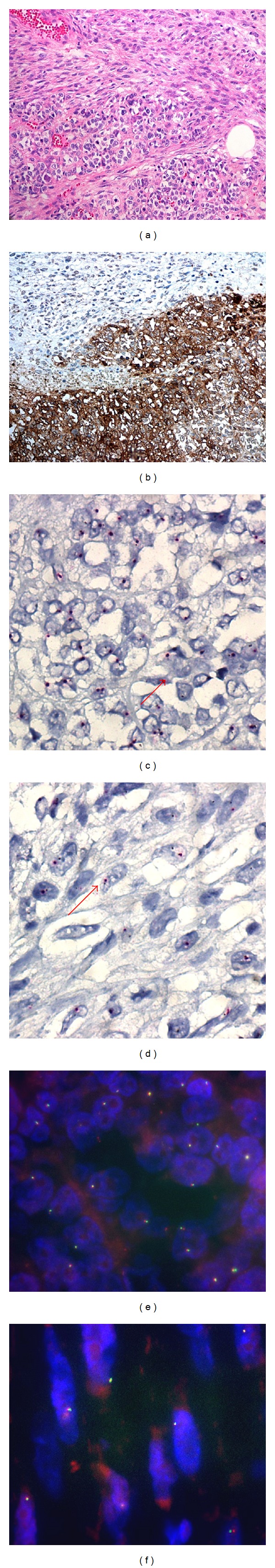
Retrospective screening of the phyllodes tumor revealed focal epithelium-like areas within the stroma composed of pleomorphic cells (panel (a), lower part of the image) that strongly expressed CD61 (panel (b)) and which were sharply demarcated from the conventional spindle cell component of the tumor (upper part of panels (a) and (b)). Chromogenic in situ hybridization of centromere 17 (red nuclear signal, arrows) revealed monosomy 17 in both the epithelial (panel (c)) and the spindle cells (panel (d)) of the tumor. Also, fluorescent fusion probes flanking 13q14 showed only one copy per nucleus (panels (e) and (f)) in both tumor components.

**Table 1:** Immunophenotypic and cytogenetic characteristics.

Tumor	Immunophenotype	Cytogenetic findings
Phyllodes tumor	CD99+, CD56+, desmin+, actin+, CD61+, factor VIII+, CD34+, CD31+	39–42, X, add(X)(q26~28), add(1)(q21), add3(q13), −5, −6, −8, −13, add(13)(q34), add(14)(p11), add(15)(p11), −16, −17, −19, add(19)(q13), −20, −21, −21, add(22)(q13), +*r*, +3mar[cp15]
Megakaryoblastic leukemia	CD99−, desmin−, actin−, CD34+, factor VIII+, CD31+, CD61+, CD56+	51~55, add(X)(q26), +1, add(3)(q11), +7, add(13)(q34)x2, add(16)(p13), inc[cp11]/46, XX[8]
